# Supplementary material for: Complexome profiling of the Chlamydomonas psb28 mutant reveals TEF5 as an early PSII assembly factor
Source: Plant Cell. 2025 Mar 17;37(6):koaf055. doi: 10.1093/plcell/koaf055 (PMC12164590; doi:10.1093/plcell/koaf055)
Supplement: koaf055_Supplementary_Data [file koaf055_supplementary_data.zip › psb28_interactive.zip!/plots/intensity/qualityPlots/peptides/FAP295_Cre16.g663200.t1.1.html]

### Cre16.g663200.t1.1

Trivial name: FAP295  
SeqCoverage [median]: 17.30  
Mass: 79.66 kDa  
  
 

| Sequence | MissedCleavage | Mass [Da] | wtIntensity | muIntensity | Score |
| --- | --- | --- | --- | --- | --- |
| DLKPENVFIDASGYVK | 1 | 1793.92 | 536.81 | 0.00 | 46.32 |
| EGEAIVYQNTPGGQR | 0 | 1617.77 | 0.00 | 411.60 | 162.68 |
| LDSQVQR | 0 | 844.44 | 227.99 | 2034.81 | 177.71 |
| LVDAFEENTYQAGQK | 0 | 1711.81 | 0.00 | 420.43 | 103.22 |
| MATVEAYTK | 0 | 1012.49 | 0.00 | 818.33 | 78.52 |
| MGMLNAR | 0 | 791.38 | 295.54 | 0.00 | 75.31 |
| MLQGMSEDLAR | 0 | 1249.58 | 366.79 | 328.35 | 114.50 |
| SNQVVAQK | 0 | 872.47 | 321.20 | 0.00 | 116.37 |
| VIVEGDKGDLFYIVK | 1 | 1693.93 | 0.00 | 3575.71 | 57.14 |
|  |  |  | 1748.3 | 7589.2 |  |
